# Supplementary material for: Triplex-forming properties and enzymatic incorporation of a base-modified nucleotide capable of duplex DNA recognition at neutral pH
Source: Nucleic Acids Res. 2021 Jul 7;49(13):7256–66. doi: 10.1093/nar/gkab572 (PMC8287925; doi:10.1093/nar/gkab572)
Supplement: gkab572_Supplemental_File [file gkab572_supplemental_file.pdf]

SUPPLEMENTARY MATERIAL

Triplex-forming properties and enzymatic incorporation of a base-modified nucleotide capable of duplex DNA recognition at neutral pH

David A. Rusling

School of Biological Sciences, University of Southampton, Southampton, Hampshire, SO17 1BJ, UK

Telephone: +44 2380 594 373

Email: d.a.rusling@soton.ac.uk

ADDITIONAL MATERIALS AND METHODS

Restriction endonuclease (REase) protection assay: TFO binding to plasmid DNA was determined by a REase protection assay. The same recombinant plasmid generated for DNase I footprinting was used in these experiments. Samples were prepared in CutSmart® buffer (50 mM potassium acetate, 20 mM tris-acetate, 10 mM magnesium acetate, 100 ug/ml BSA, at pH 7.9) (New England Biolabs). Final plasmid concentration was *ca.* 5 nM and final TFO concentrations varied between 0.1 and 10 µM depending on the experiment. Samples were prepared in a total volume of 20 µL and left to incubate overnight at 4 °C. Digestion was carried out by adding 1 µL BsmFI (0.2 units; New England Biolabs) to the samples for 15 mins at 65 °C. Products of the digestion reaction were then separated on 1% (w/v) agarose gels run at 100 V for ~1 h in 40 mM tris-acetate running buffer. Gels were subjected to staining with GelRed (Biotium) and visualised using a Gel Doc imaging system.

Circular dichroism: CD spectra of the triplexes formed with the duplex targets shown in Figure S1f,g were determined using a JASCO J-710 spectropolarimeter. Oligonucleotides were dissolved in 50 mM sodium acetate containing 10 mM $MgCl_2$ at pH 5.0, or 40 mM tris acetate containing 10 mM $MgCl_2$ at pH 7.5 and 9.0. Final concentration of the triplex was 10 µM in a total volume of 300 µL. The complexes were first heated to 95 °C for 5 min, slowly cooled to room temperature, and then left at 4 °C to equilibrate for 16 h. Spectra were collected between 320–200 nm, at 100 nm/min, 1 s response time, 1 nm bandwidth in Hellma® synthetic quartz cuvettes with a 1 mm pathlength. Each spectrum was accumulated five times, smoothed and the spectrum of the buffer was subtracted.

a
5′-TTZTTTZTTZTZT

b
5′-TTZTTTZZZTTTT

c
5′-CAACTGCGCTGGATCGGCCTTZTTTZTTZTZT

d
5′-CAACTGCGCTGGATCGGCCTTZTTTZZZTTTT

e
5′-CAACTGCGCTGGATCGGCCTTZTTTZTTZTZTZZ

f
5′-AGCTTGCATGCCTGCAGGTCGACTCTAGAGGATCCCCGAGCAGCCTAAGAAAGAAGAGAGGGACATCAGGTACCGAGCT
*AACGTACGGACGTCCAGCTGAGATCTCCTAGGGGCTCGTCGGATTCTTTCTTCTCTC*CCT*GTAGTCCCATGGC-5′

g
5′-GAGCAGCCTAAGAAA**R**AAGAGAGGGACATCA
3′-CTCGTCGGATTCTTT**Y**TTCTCTCCCTGTAGT

h
5′-GAGCAGCCTAAGAAAGGGAAAACTCACATCA
3′-CTCGTCGGATTCTTTCCCTTTTGAGTGTAGT

i
5′-AAGAAAGAAGAGA
3′-TTCTTTCTTCTCT

j
5′-TTZTTTZTTZTZT
3′-AAGAAAGAAGAGA

k
5′-CAACTGCGCTGGATCGGCC
3′-GTTGACGCGACCTAGCCGGAAGAAAGAAGAGA-P-5′

l
5′-CAACTGCGCTGGATCGGCC
3′-GTTGACGCGACCTAGCCGGAAGAAAGGGAAAA-P-5′

m
5′-CAACTGCGCTGGATCGGCC
3′-GTTGACGCGACCTAGCCGGAAGAAAGAAGAGAGG-P-5′

Figure S1: Sequence of oligonucleotides used in this study. (a-b) Synthetic 13-mer TFOs containing isolated or contiguous substitutions of Z, respectively (in bold); (c-d) Enzymatically assembled oligonucleotides containing a 13-mer triplex-forming sequence with isolated or contiguous substitutions of Z, respectively (in bold); (e) Enzymatically assembled oligonucleotide containing a 15-mer triplex-forming sequence with isolated and contiguous substitutions of Z at the 3´-end of the oligonucleotide (in bold); (f) 73-mer duplex fragment used in footprinting experiments containing the embedded target sequence for the TFO containing isolated modifications (boxed). The pyrimidine-containing strand was labelled at the 3´-end and shown with an asterisk. (g-h) 31-mer duplex sequences containing an embedded target sequence (boxed) with isolated and contiguous GC base pairs, respectively. The interaction of the TFOs was examined by fluorescence melting, EMSA and CD experiments. The duplex containing isolated GC base pairs was also used for selectivity studies by positioning a single Z modification in the third strand opposite each base pair in turn (highlighted RY in bold); (i) 13-mer duplex sequence containing the target sequence for the TFO containing isolated substitutions (boxed). The interaction of the TFOs was examined by fluorescence melting. (j) 13-mer duplex formed by the association of a Z-containing oligonucleotide with its W-C complement forming ZG base pairs. The interaction of the TFO was examined by fluorescence melting experiments. (k-m) Templates used to assemble oligonucleotides containing a triplex-forming sequence with isolated or contiguous substitutions of Z by primer extension. The template strand was synthesised with a 5´-phosphate (in yellow) to allow its selective degradation by Lambda exonuclease and release of the assembled primer-TFS oligonucleotide. Assembly was assessed by an EMSA and the interaction of the TFOs examined by fluorescence melting and/or EMSA experiments.

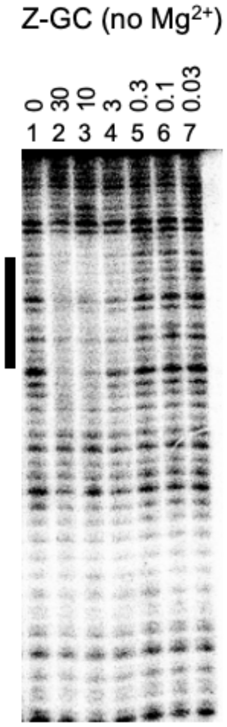

Figure S2: TFO binding in absence of magnesium. DNase I cleavage patterns for the 73-mer duplex fragment containing the embedded TFO target sequence in the absence and presence of the synthetic TFO designed to generate isolated Z-GC triplets (Figure S1a). Experiments were performed in 50 sodium acetate buffer without magnesium. TFOs were incubated with the fragment overnight at 4 °C at a final concentration of 30, 10, 3, 0.3, 0.1, 0.03 µM as indicated before digestion by the enzyme. Samples were separated on a denaturing 12% polyacrylamide gel and subjected to phosphorimaging. The pyrimidine-containing strand of the fragment was labelled with ^{32}P at its 3´-end. The TFO target sequence is shown by the boxes adjacent to each gel and was determined by comparison with bands in a Maxim-Gilbert marker lane (labelled ‘M’).

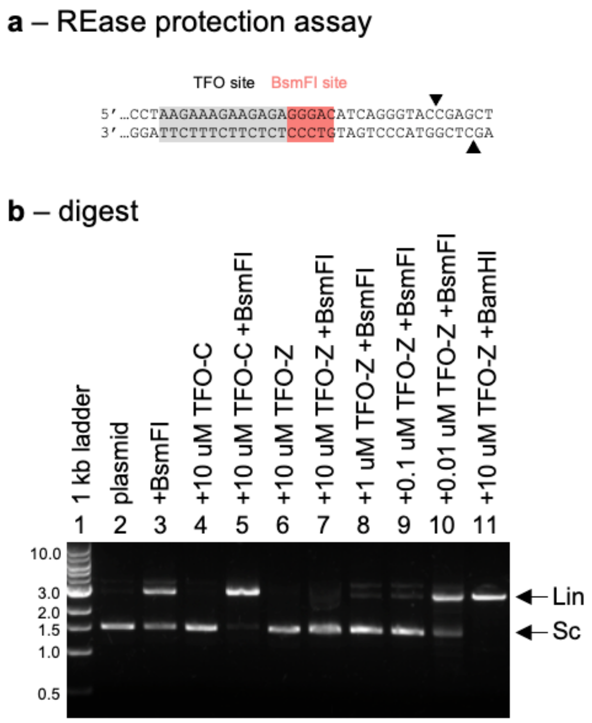

Figure S3: **Binding of TFO to plasmid DNA.** Binding of the Z-containing TFO (TFO-Z) shown in Figure S1a to its target sequence located within a supercoiled plasmid was determined by a restriction endonuclease (REase) protection assay. (a) The TFO target sequence (grey box) was located adjacent to the asymmetric recognition sequence of the Type IIS REase BsmFI (peach box). In the absence of TFO the REase cuts the supercoiled (Sc) plasmid 10 and 14 nucleotides downstream of this sequence (shown on each strand by the triangles), generating a linearised product (Lin). Whilst in the presence of the TFO the reaction is inhibited by steric occlusion of the enzyme or through structural changes propagated to the adjacent sequence; (B) Plasmid DNA was incubated with either 10, 1, 0.1 or 0.01 μM TFO (as indicated) in CutSmart® buffer (pH 7.9) overnight and the samples subsequently digested with 0.2 units of BsmFI for 15 minutes at 65 °C. The products of the reactions were separated on a 1% agarose gel alongside appropriate controls and subjected to staining with GelRed. The lack of linearisation of the plasmid in the presence of the modified TFO indicates TFO binding and protection from the enzyme (lanes 7-10). As expected, protection is concentration dependant and decreases as the TFO concentration decreases. The plasmid was also digested by BamHI in the presence of the modified TFO and no protection from digestion was observed (lanes 7-10), suggesting the oligonucleotide does not bind to other sequences in the plasmid. No protection was seen for the unmodified TFO (TFO-C) which is not likely to bind at this pH and relatively high temperature (lane 5).

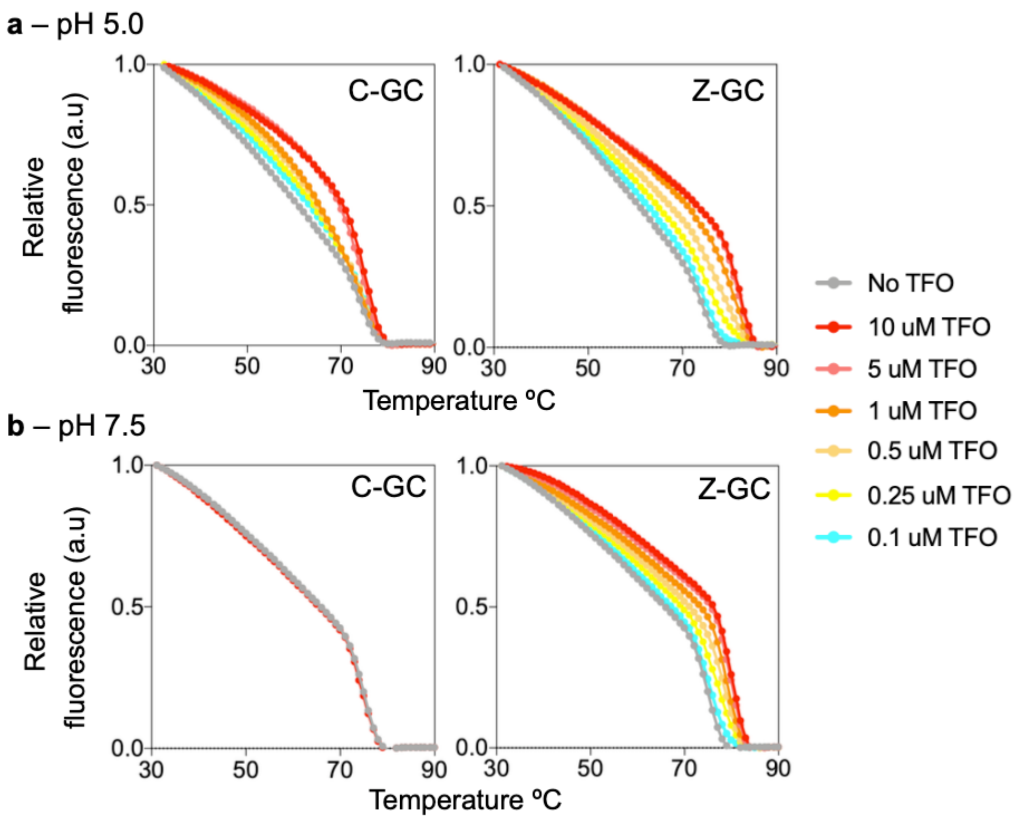

Figure S4: **Concentration dependence of TFO binding.** Fluorescence melting profiles for the triplexes containing either C^+-GC or Z-GC triplets at isolated positions at different concentrations of third strand. The target sequence was located centrally within a 31-mer duplex. Samples were prepared in sodium cacodylate buffer containing magnesium at either pH 5.0 (a) or pH 7.5 (b). The final concentration of the duplex was 1 μM and the final concentrations of the TFOs varied between 0.1 and 10 μM as indicated. The complexes were melted at a rate of 0.2 °C/min in the presence of SYBR green and the fluorescence signal recorded at 522 nm after excitation at 488 nm. Oligonucleotide sequences are shown in Figure S1a,g and the melting temperatures for the complexes in Table S1.

pH	Triplex	0	10 μM	5 μM	1 μM	0.5 μM	0.25 μM	0.1 μM
5.0	C^+-GC	73.0	74.2	73.6	73.2	73.4	73.4	73.5
	Z-GC	73.5	81.8	81.8	81.1	77.9	75.3	74.5
7.5	C-GC	75.0	74.8	-	-	-	-	-
	Z-GC	75.0	80.1	79.7	79.1	78.6	77.0	75.2

Table S1: Concentration dependence of TFO binding. T_m values (ºC) calculated for the triplexes containing either C^+-GC or Z-GC triplets at isolated positions at different concentrations of third strand.

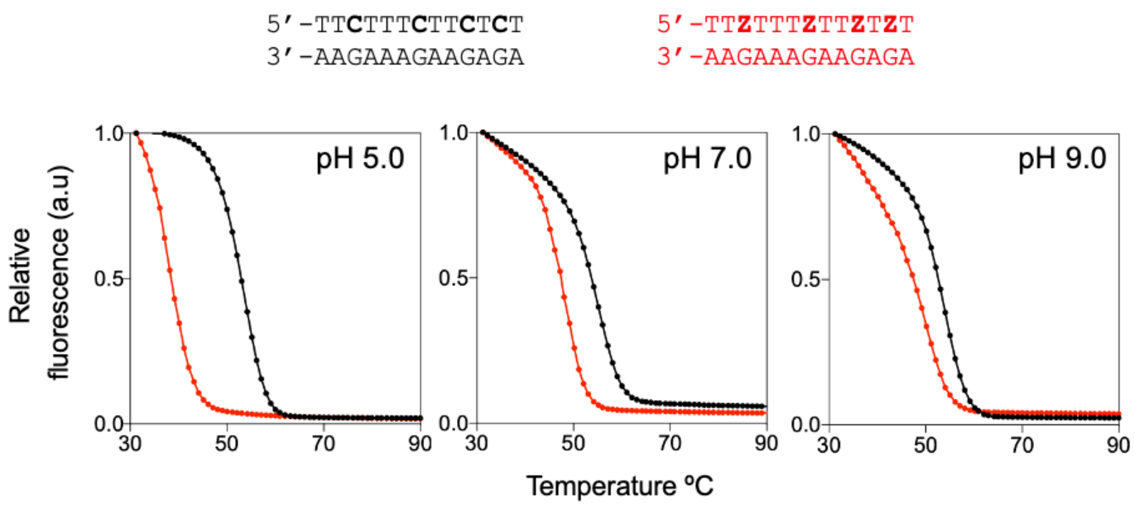

Figure S5: pH dependence of Z-G base pairs. Fluorescence melting profiles for the duplexes containing either CG (black) or ZG base pairs (red) at different pH values. Samples were prepared in sodium cacodylate buffer containing magnesium at either pH 5.0, pH 7.0 or pH 9.0 as indicated. The final concentration of the duplexes was 1 μM. The complexes were melted at a rate of 0.2 °C/min in the presence of SYBR green and the fluorescence signal recorded at 522 nm after excitation at 488 nm. Sequences are shown in Figure S1j and the melting temperatures for the complexes in Table S2.

	pH 5.0	pH 6.0	pH 7.0	pH 8.0	pH 9.0
CG duplex	53.5	55.0	55.3	54.3	54.0
ZG duplex	<40	42.4 (-12.6)	48.1 (-7.2)	50.3 (-4.0)	49.8 (-4.2)

Table S2: pH dependence of Z-G base pairs. T_m values (ºC) calculated for the duplexes containing either CG or ZG base pairs at different pH values. Values in parenthesis are the delta T_m values between ZG-containing and CG-containing duplexes.

	pH 5.0	pH 7.5	pH 9.0
Duplex	50.6	52.7	51.4
Synthetic TFO	66.7	66.1	64.1
Assembled TFO	66.1	64.7	64.0

Table S3: Thermal stability of triplexes determined for synthetic and template assembled TFOs containing the Z nucleobase. T_m values (ºC) calculated at different pH values for the interaction of the TFOs shown in Figure S1a,c and duplex shown in Figure S1i. Comparison of T_m data between the synthetic and template-made TFOs reveals little difference in affinity of the oligonucleotides; misincorporation of the wrong nucleotides in the TFO during assembly would have drastically reduced TFO affinity.

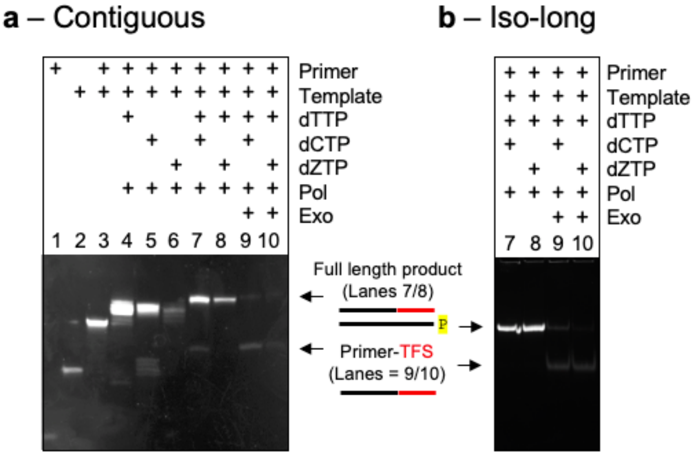

Figure S6: Template-directed assembly of oligonucleotides containing different triplex-forming sequences. (a) Assembly of the oligonucleotide containing a 13-mer TFS containing contiguous Z modifications (Figure S1d); (b) Assembly of the longer oligonucleotide containing a 15-mer TFS containing both isolated and contiguous Z modifications (Figure S1e). In each case EMSAs are included to show the products of the extension and digestion reactions. The composition of each sample is shown above each lane of the gel. Final concentration of the strands and dNTPS was 5 μM and 100 μM, respectively. Extension reactions were performed for 2 hrs at 72 °C with 2 units of Therminator® polymerase, whilst digestion reactions were performed for 2 hrs with 10 units of Lambda exonuclease at 37 °C. The complexes were then separated on a 20% non-denaturing polyacrylamide gel and subjected to post-staining with GelRed.

9

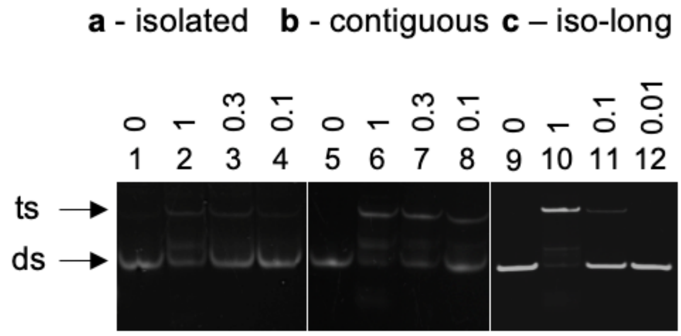

Figure S7: Triplex formation at neutral pH with enzymatically assembled TFOs. EMSAs for the oligonucleotides containing isolated (a) and contiguous (b) Z-GC triplets, as well as for a longer triplex that contains an additional two Z-GC triplets at the 3´-end (C). TFO sequences are shown in Figures S1c,d,e, respectively. The target sequence was located centrally within a 31-mer duplex. Oligonucleotides were prepared in tris acetate buffer containing magnesium at pH 7.5. The final concentration of the duplexes were 1 μM and the final concentrations of the TFOs were varied between 0.1 μM and 1 μM as indicated. The complexes were separated on a 20% non-denaturing polyacrylamide gel in tris acetate running buffer containing magnesium. Gels were subjected to post-staining with GelRed. Arrows indicate the double-stranded (ds) or triple-stranded (ts) complexes.

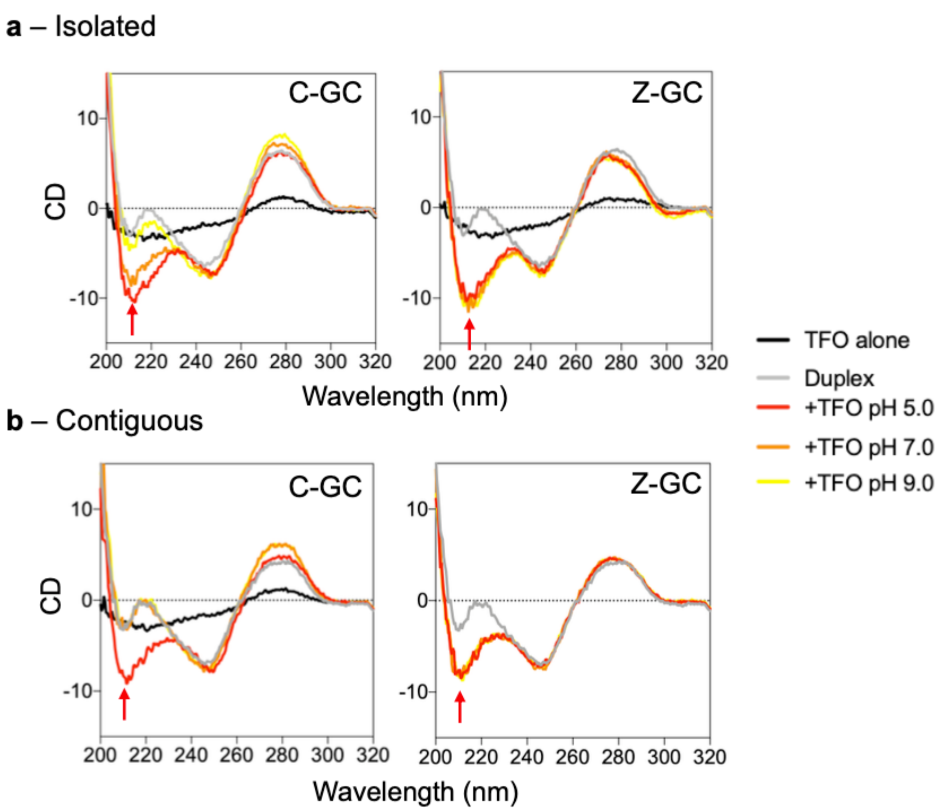

Figure S8: **CD spectra of triplexes.** CD spectra for the triplexes containing either C-GC or Z-GC triplets at isolated (a) or contiguous (b) positions. The target sequence was located centrally within a 31-mer duplex. Oligonucleotides were annealed to 20 ºC at a final concentration of 5 uM in buffer containing magnesium at the pH value indicated. Spectra were collected between 320–200 nm and the spectrum of the buffer subtracted. A negative peak at 210 nm is indicative of triplex formation (shown by the arrow) due to the A-like nature of the helices.

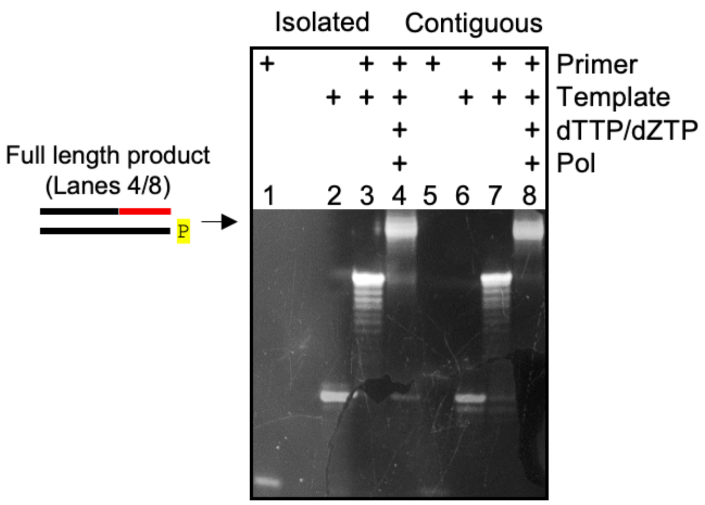

Figure S9: Template-directed assembly of TFOs containing isolated or contiguous Z modifications from an RNA primer. EMSA showing the products of the extension reactions. The composition of each sample is shown above each lane of the gel. Final concentration of the strands and dNTPS was 5 μM and 100 μM, respectively. Extension reactions were performed for 2 hrs at 72 °C with 2 units of Therminator® polymerase. The complexes were then separated on a 20% non-denaturing polyacrylamide gel and subjected to post-staining with GelRed. Full length products are seen for both templates showing extension is possible from an RNA primer.
